# Electronically Tunable Memristor Emulator Implemented Using a Single Active Element and Its Application in Adaptive Learning

**DOI:** 10.3390/s23031620

**Published:** 2023-02-02

**Authors:** Sadaf Tasneem, Pankaj Kumar Sharma, Rajeev Kumar Ranjan, Fabian Khateb

**Affiliations:** 1 Electronics Engineering Department, Indian Institute of Technology (Indian School of Mines) Dhanbad, Dhanbad 826004, Jharkhand, India; 2 Department of Microelectronics, Brno University of Technology, 601 90 Brno, Czech Republic; 3 Faculty of Biomedical Engineering, Czech Technical University in Prague, nám. Sítná 3105, 272 01 Kladno, Czech Republic; 4 Department of Electrical Engineering, University of Defence, Kounicova 65, 662 10 Brno, Czech Republic

**Keywords:** memristor emulator, DVCC, pinched hysteresis loop (PHL), Monte Carlo, adaptive learning

## Abstract

In recent times, much-coveted memristor emulators have found their use in a variety of applications such as neuromorphic computing, analog computations, signal processing, etc. Thus, a 100 MHz flux-controlled memristor emulator is proposed in this research brief. The proposed memristor emulator is designed using a single differential voltage current conveyor (DVCC), three PMOS transistors, and one capacitor. Among three PMOS transistors, two transistors are used to implement an active resistor, and one transistor is used as the multiplier required for the necessary memristive behaviors. Through simple adjustment of the switch, the proposed emulator can be operated in incremental as well as decremental configurations. The simulations are performed using a 180 nm technology node to validate the proposed design and are experimentally verified using AD844AN and CD4007 ICs. The memristor states of the proposed emulator are perfectly retained even in the absence of external stimuli, thereby ascertaining the non-volatility behavior. The robustness of the design is further analyzed using the PVT and Monte Carlo simulations, which suggest that the circuit operation is not hindered by the mismatch and process variations. A simple neuromorphic adaptive learning circuit based on the proposed memristor is also designed as an application.

## 1. Introduction

In 1971, Leon Chua speculated the fourth fundamental passive element [1], presenting the essential relation between charge and flux, and called it a memristor. Later, in 2008, Strukov et al. [2] developed the first successful fabrication of a memristor using TiO_2_ at Hewlett Packard (HP). This fabricated memristor attracted many researchers across the globe, and since then, many attempts have been made to further explore the domain of memristors. Typically, memristors store information in the form of resistance states [3,4]. These states are maintained even in the absence of applied input which is regarded as an essential criterion for non-volatility. Memristors can be used in many applications such as chaotic circuits, adaptive filters, programmable analog circuits, non-volatile memories, neuromorphic circuits, and many more analog circuits [5,6,7,8]. However, as memristive technologies are still considered fairly recent technology, their commercialization at a larger scale remains elusive. Moreover, owing to their fabrication complexity, the physical realization of these memristors poses a lot of challenges. Hence, to overcome these challenges with an aim to exploit its potential uses, many researchers came up with memristor emulators capable of mimicking the attributes of a real memristive device. Several memristor models have been implemented using various analog building blocks (ABB), such as second-generation current conveyors (CCII) [9,10], operational transconductance amplifiers (OTA) [11,12,13], voltage differencing current conveyors (VDCC) [14], current backward transconductance amplifiers (CBTA) [15], current conveyor transconductance amplifiers (CCTA) [16], and voltage differential buffered amplifiers (VDBA) [17]. Similar blocks, i.e., voltage differencing transconductance amplifiers (VDTA), were utilized in refs. [18,19], with the emulators operating up to 50 MHz. Gupta et al., in ref. [20], designed a current differencing transconductance amplifier (CDTA) and an OTA-based memristor emulator with an operating frequency range of 600 kHz to 2 MHz for application in the current mode filter. A differential voltage current conveyor transconductance amplifier (DVCCTA) [21] based a memristor design in incremental as well as decremental configuration and operating up to 12.8 MHz was also proposed. Sagar and his team also presented a current follower transconductance amplifier (CFTA)-based [22] resistor-less emulator model. Furthermore, apart from ref. [20], mixed ABB has also been employed in designing various memristor models, such as in refs. [23,24,25,26]. Along with ABB, these emulators also incorporate a few passive elements such as resistors and capacitors. Some researchers developed a MOS-based memristor emulator [27,28], but those designs had certain limitations, such as a lack of tunability features. A differential voltage current conveyor (DVCC) is an active element which is an extension of the widely used CCII block. DVCC emerge as a useful choice in applications where differential inputs or two high-input impedance terminals are required. Thus, we chose DVCC to implement the proposed memristor emulator. Therefore, this article proposes a flux-based memristor emulator operating up to 100 MHz using only one DVCC as an active element. The proposed emulator also contains an electronically tunable active resistor, one grounded capacitor, and one PMOS. The active resistor was designed using two PMOS transistors. Simulations of the proposed emulator were conducted using a 180 nm CMOS process in the Analog Design Environment (ADE) of Cadence Virtuoso Software. The experimental results have also been presented using commercially available AD844AN and CD4007 ICs to validate the theoretical propositions. Further, a summary of the various existing designs and highlights of the proposed design are listed in Table 1. A few significant merits of this new memristor model proposed herein are listed as follows:

1. A single active block, i.e., DVCC, is used to implement the memristive behavior that comprises only one capacitor as a passive element.
2. The operating frequency achieved here is the highest (up to 100 MHz) when compared to other recently available emulator models.
3. The tunability is an additional advantage achieved using two PMOS serving as an active resistor.
4. Lastly, the transistor count is the least among all the available designs, i.e., 15 transistors.

## 2. Differential Voltage Current Conveyor (DVCC) Block

**Figure 1:**
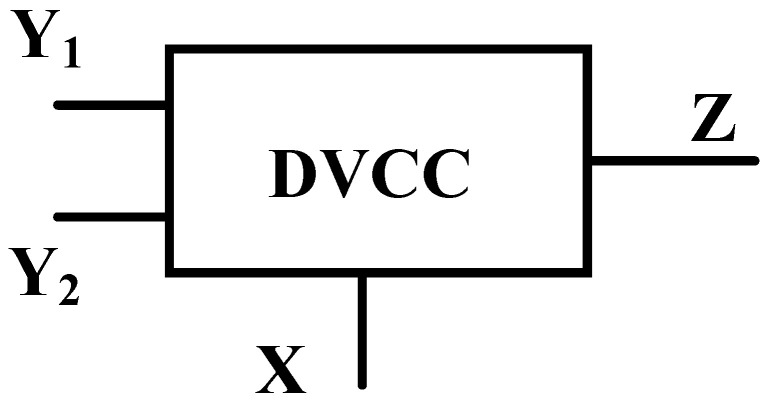
DVCC block diagram.

The DVCC element is a widely used active analog signal processing block. It is the advanced extension of CCII, which provides both differential voltage at the input side and current copying capability at the output. The DVCC has four terminals *Y*_1_, *Y*_2_, *X*, and *Z*. Among these four terminals, the *Y*_1_ and *Y*_2_ terminals have high input impedance, *X* has low input impedance, and *Z* has high output impedance. The block diagram of DVCC is introduced in Figure 1. 

**Figure 2:**
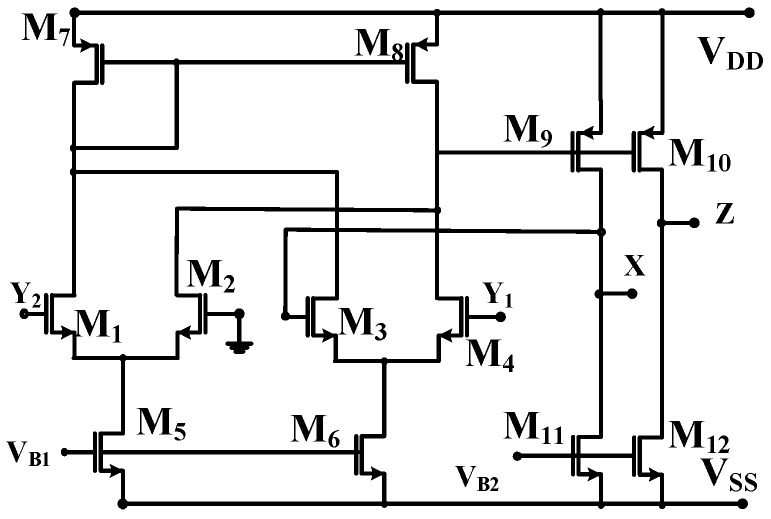
MOSFET realization of DVCC.

The port relation of DVCC is listed in Equation (1). The MOSFET realization of DVCC is shown in Figure 2, where the differential voltage of terminals *Y*_1_ and *Y*_2_ appears at terminal *X*. The current through terminals *X* and *Z* is equal.

(1) $$I_{Y1}=I_{Y2}=0,V_{X}=V_{Y1}-V_{Y2},I_{Z}=I_{X}.$$

## 3. Proposed Memristor Emulator Design

**Figure 3:**
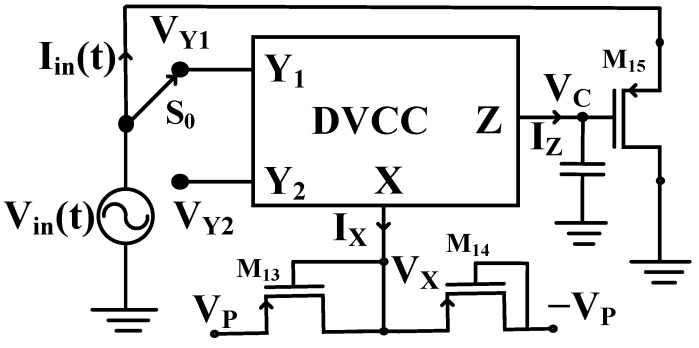
The flux-controlled proposed memristor emulator.

The memristor essentially presents the essential relation between charge and flux and turns out to be the fourth fundamental circuit element. The proposed design contains one DVCC, one capacitor, and three PMOS transistors. Among three PMOS transistors, two transistors are used to obtain an active resistor, and one PMOS transistor, along with the capacitor, is used to develop memristor emulator functionality. Alternatively, the variable flux caused by the capacitor voltage is responsible for creating the variable resistance states, which shows the hysteretic memristive behavior. In this article, we propose a flux-controlled memristor emulator. The proposed emulator design is shown in Figure 3. The equivalent resistance (*R_eq_*) can be adjusted using control voltage *V_P_*, as described in Equation (2).

(2) Req=12μpCox(W/L)13,14(VP−VTH).

### 3.1. Mathematical Analysis of Memristor Emulator

The mathematical analyses of the proposed emulator are described below: 

From the DVCC port relationship, we have

(3) $$V_{X}=V_{Y1}-V_{Y2}.$$

Input voltage is exerted at terminal *Y*_1_ or *Y*_2_. Therefore, the *V_X_* value can be expressed as:

(4) $$V_{X}=\pm V_{in}\left(t\right).$$

The current equation at *X* terminal is:

(5) IX=±Vin(t)Req=IZ.

The capacitor voltage *V_C_* is equal to *V_Z_* and can be calculated as:

(6) VZ=VC=±1C∫(±Vin(t)Req)dt=±ϕ(t)CReq.

The capacitor voltage $V_{C}\;$ drives the MOS M_15_. Ignoring the output resistance of the PMOS, conductance ($g_{m}$) at the input port can be achieved as follows:

(7) Iin(t)Vin(t)=gm=μpCox(WL)15(VG−VS−VTP),

where $C_{ox}$ is the gate-oxide capacitance per unit area, $\mu_{p}$ is the mobility, *W/L* is the aspect ratio of MOS M_15_, *V_G_* and *V_S_* are the gate and source voltages of the M_15_ transistor, respectively, and $V_{TP}$ is the threshold voltage of the PMOS transistor, which has a negative value. The input signal is exerted at the source of the transistor, and the DC value of the input voltage is zero. Therefore, $g_{m}$ can be rewritten as:

(8) gm=Iin(t)Vin(t)=μpCox(WL)15(VG−VTP).

The gate voltage of transistor M_15_ is a function of input flux (φ(t)) equal to the capacitor voltage *V_C_* expressed in Equation (6) and can be rewritten as:

(9) $$V_{G}=V_{C}=\pm \frac{\phi \left(t\right)}{CR_{eq}}.$$

The conductance value of transistor M_15_ from Equation (8) can be modified after setting the gate voltage from Equation (8) as:

(10) Iin(t)Vin(t)=μpCox(WL)15(±ϕ(t)CReq−VTP).

The memductance equation of the proposed emulator design is provided as:

(11) W(ϕ(t))=Iin(t)Vin(t)=−μpCox(WL)15︸1stPartVTP±μpCox(WL)15ϕ(t)CReq︸2ndPart.

Equation (11) has two parts, the first part is the time-independent part, and second part is the time-dependent part which depends on input flux. Therefore, the proposed memristor emulator is the flux-controlled memristor. The positive and negative sign of the second part of the memductance equation indicates that the memristor emulator works in both decremental and incremental configurations depending upon the position of the switch “S_0_”.

### 3.2. Frequency Response Analysis

To test the dependency of the design on the operating frequency, we applied a sinusoidal input:

(12) $$V_{in}\left(t\right)=A_{m}\sin\omega t.$$

The input flux can be obtained by integrating input voltage as:

(13) ϕ(t)=∫Amsinωtdt=Amcos(ωt−π)ω.

By substituting the flux value from Equation (13) in Equation (11), we can obtain the memductance value of the proposed memristor emulator as:

(14) W(ϕ(t))=Iin(t)Vin(t)=−μpCox(WL)15︸1stPartVTP±μpCox(WL)15Amcos(ωt−π)ωCReq︸2ndPart.

From Equation (14), it is visible that it has two parts: the first part is the time-invariant part, and the second part is the time-variant part. Since it is evident from Equation (14) that the memductance holds an inverse relation with frequency, the second part decreases with increasing frequency. Subsequently, with frequency tending to infinity, the second part is completely lost, and only the first part is retained, suggesting that the non-linear behavior of the emulator transforms to a single-valued linear resistor characteristic. Hence, it can be stated that the memristor at high frequency fails to contain any hysteresis, eventually losing its memory-storing capacity and behaving like a simple resistor.

The parameter ‘α’ is basically the ratio of the modulus values of the amplitude of a time-dependent part to that of a time-independent part and can be calculated as:

(15) $$\alpha =\frac{A_{m}}{\omega CR_{eq}\left|V_{TP}\right|}=\frac{A_{m}}{2\pi fCR_{eq}\left|V_{TP}\right|}=\frac{1}{\tau f}=\frac{T}{\tau },$$

where *T* and *τ* are time period and time constant, respectively. The time constant (τ) is written as:

(16) $$\tau =\frac{2\pi fCR_{eq}\left|V_{TP}\right|}{A_{m}}.$$

Based on Equation (15), we can conclude that: when f approaches infinity, α approaches zero, and the linear time-invariant conductance part dominates the memristor behavior.

## 4. Discussion

**Figure 4:**
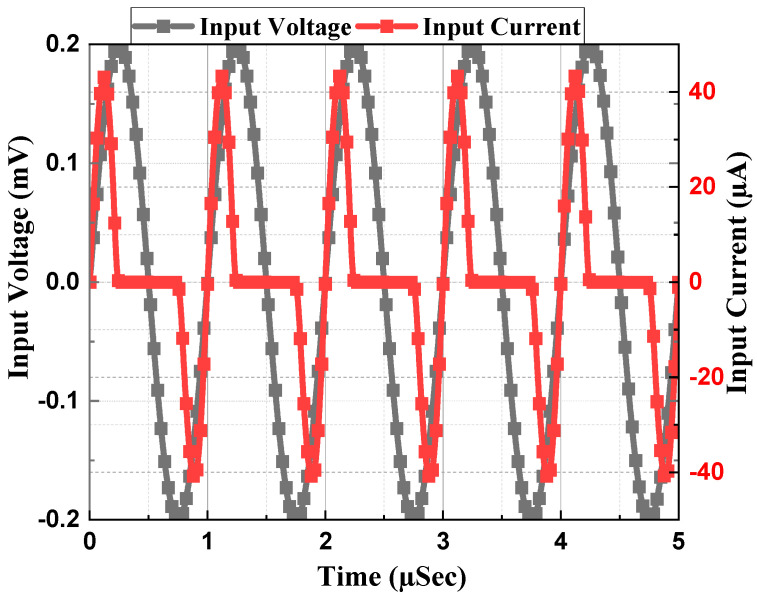
Transient Analysis at 1 MHz with 15 pF Capacitor.

**Figure 5:**
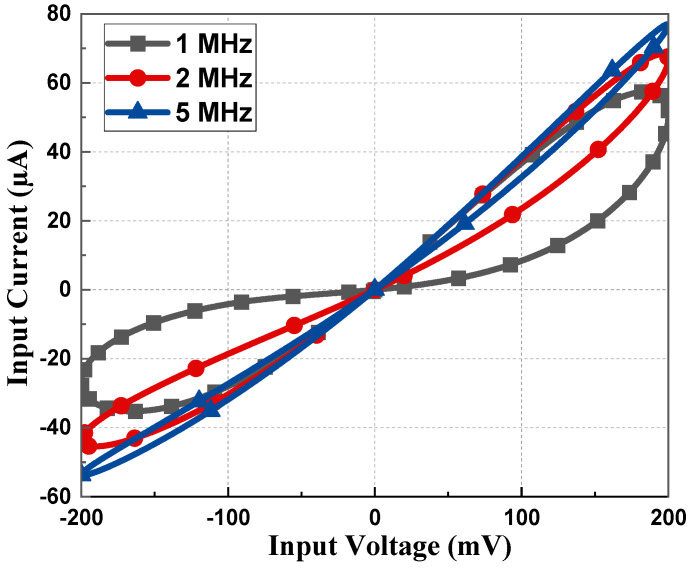
PHL at 1, 2, and 5 MHz with 50 pF capacitor value for incremental configuration.

**Figure 6:**
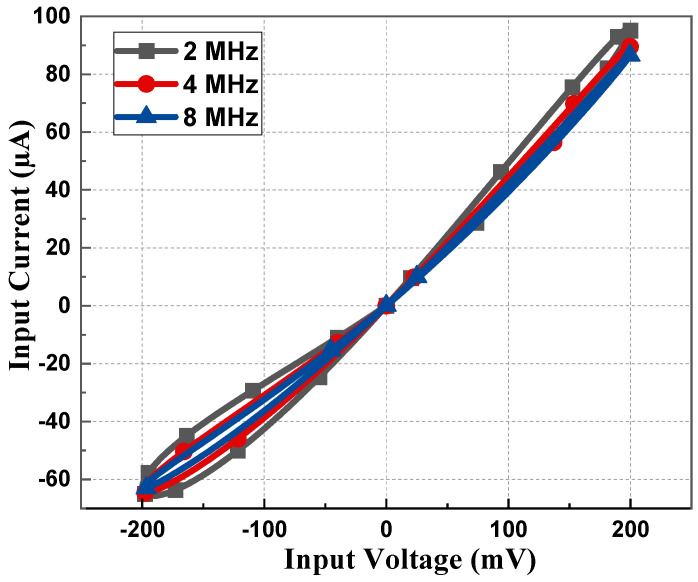
PHL at 2, 4, and 8 MHz with 50 pF capacitor value for decremental configuration.

The memristor emulator based on a single DVCC presented in Figure 3 was simulated using cadence virtuoso software with 180 nm CMOS technology. The DC supply for the DVCC was chosen as *V_DD_* = −*V_SS_* = 1.25 V with the biasing voltages as *V_B_*_1_ = 0.8 V and *V_B_*_2_ = 0.4 V. The aspect ratio of all the transistors is listed in Table 2. Most of all PMOS is connected to *V_DD_*, and for NMOS, it is connected to *V_SS_*. To design an active resistor, *V_p_* is chosen as 0.5 V. As the memristor is a passive element, the current becomes zero when the input voltage is zero, as depicted in Figure 4. Transient analysis runs over five cycles at 1 MHz frequency and 15 pF capacitor value. The memristor PHL in the V-I plane at several frequencies for both incremental and decremental configurations are presented in Figure 5 and Figure 6. These PHLs clearly depict the distinguishable resistance states, i.e., high resistance and low resistance states at lower frequencies.

**Figure 7:**
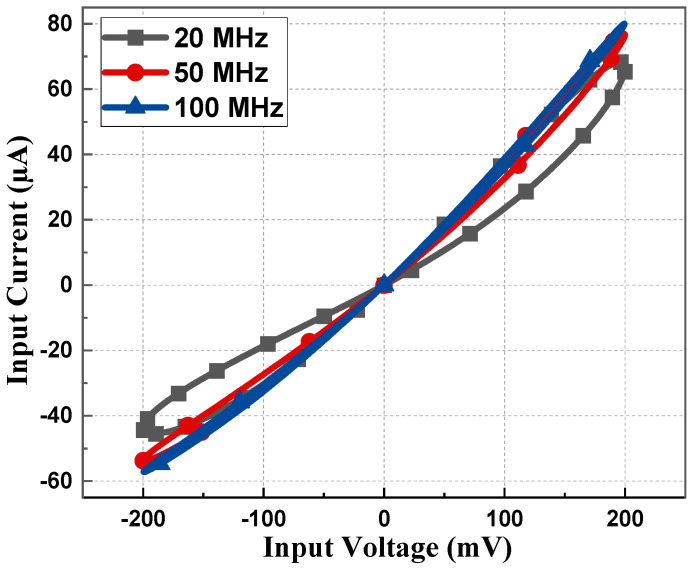
PHL at 20, 50, and 100 MHz with 5 pF capacitor value for incremental configuration.

The PHL of the proposed memristor and its resistance states at considerably higher frequencies are included in Figure 7. From Figure 7, it can be deduced that the proposed emulator design operates up to 100 MHz. It is clearly observed from Figure 5, Figure 6 and Figure 7 that the non-linear nature of the memristor circuit starts changing as the frequency changes. With an increase in frequency, the hysteresis behavior starts to cease until it eventually vanishes at high frequency, i.e., greater than 100 MHz in this case. Such a behavior of the loop is attributed simply to the frequency that controls the second part of Equation (14).

**Figure 8:**
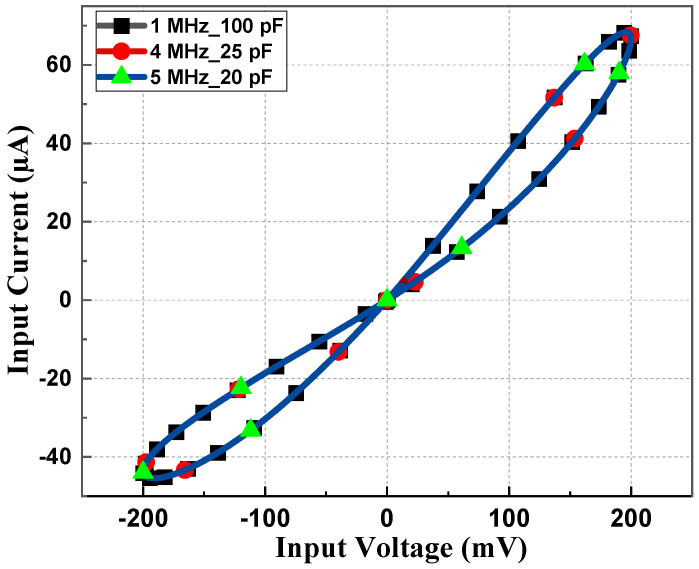
PHL at constant *fC* product.

Figure 8 shows the current–voltage curve for constant frequency and capacitance product at 1 MHz, 4 MHz, and 5 MHz frequencies with 100 pF, 25 pF, and 20 pF capacitor values, respectively. In all the aforementioned values of the frequency and capacitor pair, the frequency–capacitance product remains unchanged, hence there is no deviation in the PHL of Figure 8.

**Figure 9:**
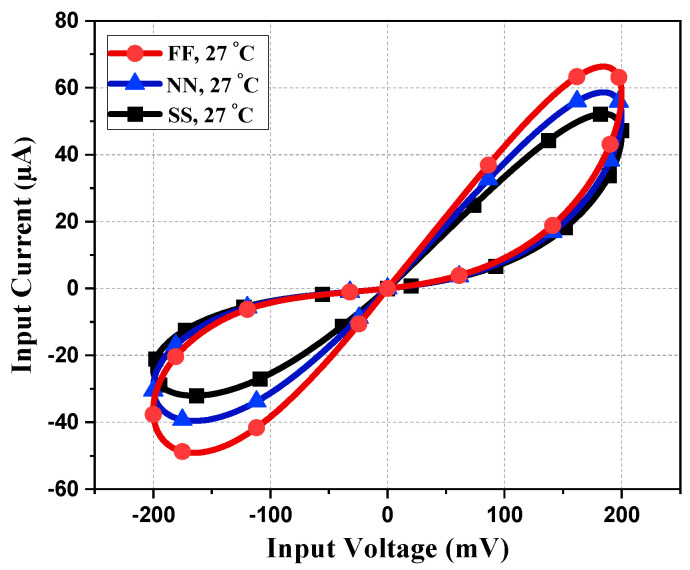
PHL at different process corners.

**Figure 10:**
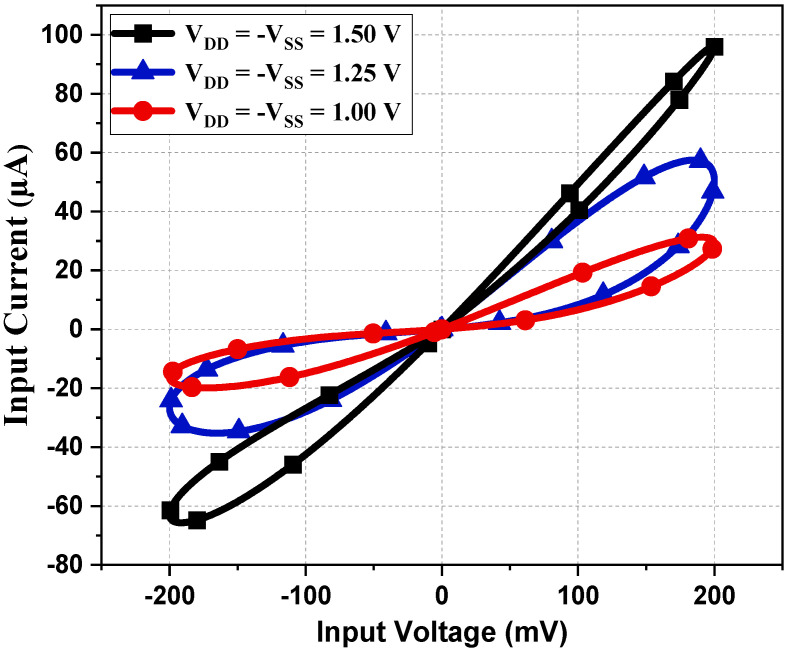
PHL at different supply voltages.

**Figure 11:**
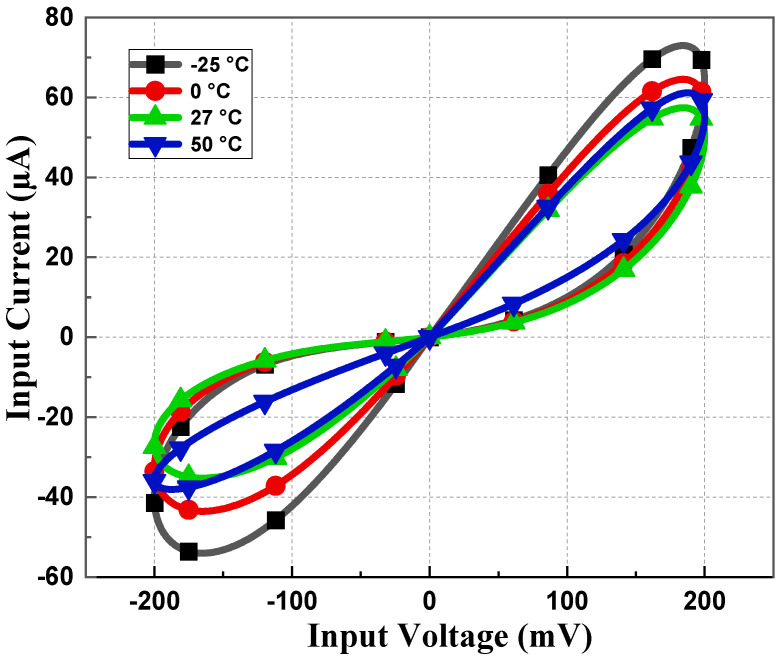
PHL at different temperatures at 5 MHz with 10 pF capacitor value.

**Figure 12:**
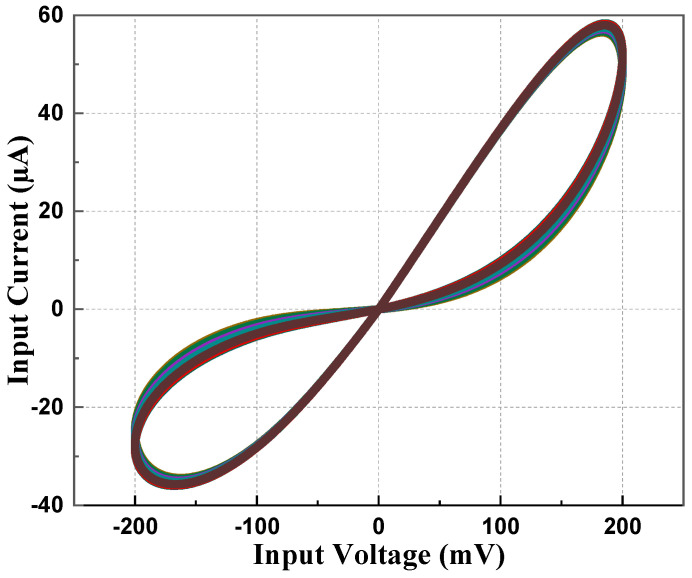
Monte Carlo simulation at 5 MHz with 10 pF.

To observe the robustness of the proposed memristor, the PHL at different process corners is obtained at 27 °C of temperature. From Figure 9, it is evident that the loop area of PHL for the slow–slow (SS) corner is less compared to the fast–fast (FF) corner, justifying the fact that the current flow in the SS corner will be less than the FF corner. Memristor behavior for different supply voltages is observed and presented in Figure 10. It is observed that the slope of PHL changes as supply changes, but the memristance nature remains intact. Figure 11 displays the proposed design works for a wide range of temperatures. From Figure 11, it is evident that the current through the memristor emulator decreases with increased temperature. The Monte Carlo simulation was carried out for 200 runs with 5% passive element variation to check the ambiguity and robustness of the proposed design. It can be witnessed in Figure 12 that the workability of the proposed design is quite stable and can operate within a tolerable limit.

**Figure 13:**
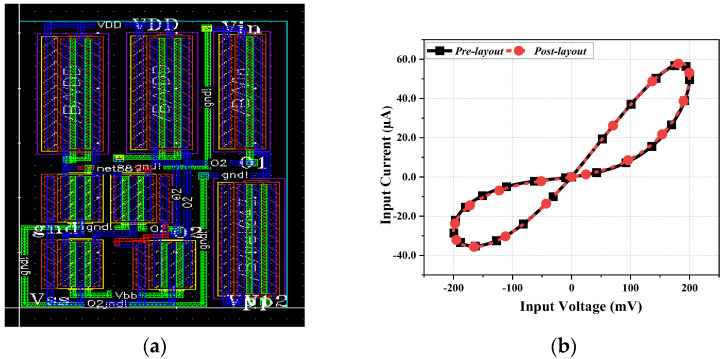
(**a**) Layout of proposed memristor design. (**b**) Pre-layout and post-layout PHL.

The layout of the proposed memristor design, excluding the capacitor (C), is depicted in Figure 13a. It utilizes a chip area of 17 µm × 26 µm. The comparison between pre-layout and post-layout simulation in the I-V plane is depicted in Figure 13b. It is observed that there is a slight deviation in the simulation result due to the presence of parasitic elements, which became evident when we extracted the parameters of the layout. The power consumption of the proposed memristor is 7.5 µW which is quite low, making this design suitable for low-power applications.

**Figure 14:**
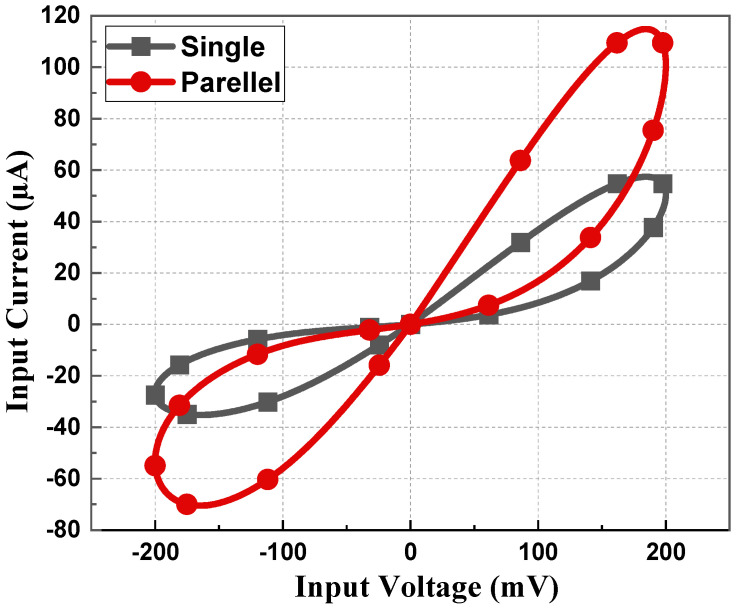
PHL for parallel and single memristor at 5 MHz with 10 pF capacitor.

**Figure 15:**
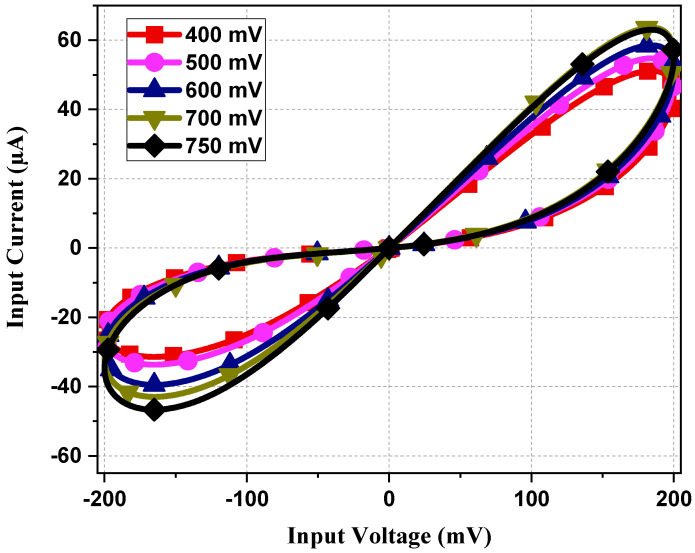
PHL for different values of *V_P_* at 5 MHz with 10 pF capacitor value.

Various connections of the proposed design are tested in Figure 14. Parallel connections have less memristance value and conduct more current than single memristors. From Figure 14, it is clearly observable that parallel connections have a bigger loop and a large current value compared to single memristor PHLs. Figure 15 demonstrates the PHL behavior for different values of *V_P_*. From Figure 15, it can be inferred that the proposed design exhibits a tunability feature by varying values of *V_P_* voltage; resistance value changes and thus PHL area changes. 

**Figure 16:**
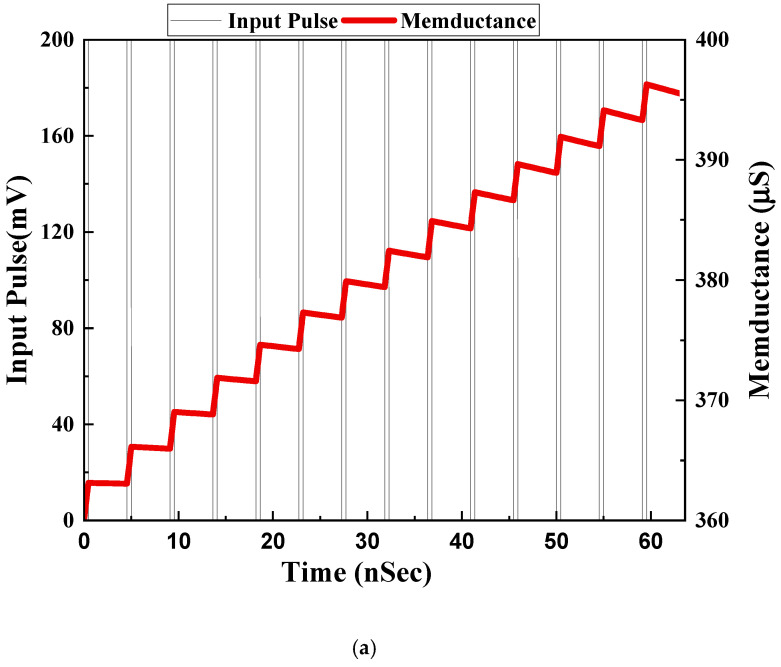

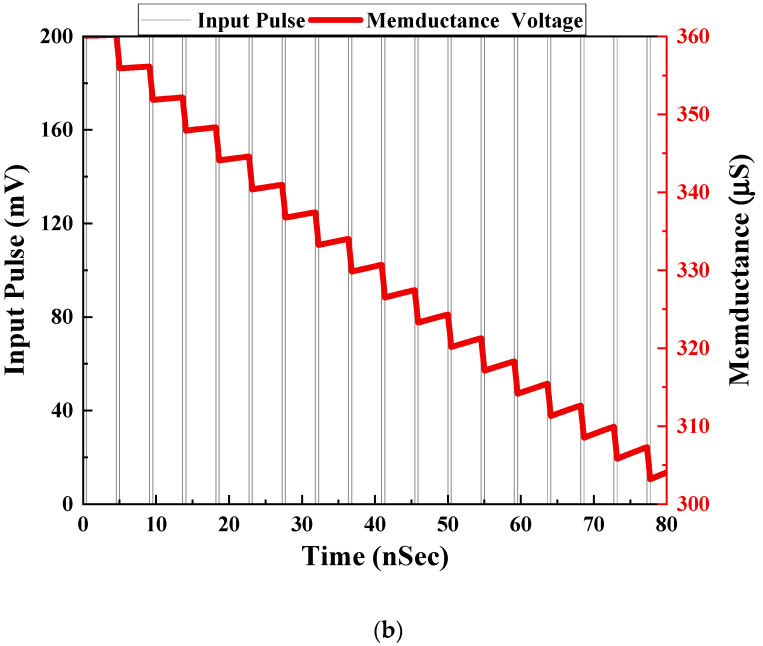
Memductance variation with input pulse for (**a**) incremental configuration and (**b**) decremental configuration.

To reflect the memory effect of the memristor, the non-volatility test was performed for both configurations. To achieve non-volatility, we took a 200-mV pulse with a 5 ns period and 0.5 ns ON time. The capacitor value taken for the non-volatility test was 5 pF. Figure 16a,b shows the non-volatility test for incremental and decremental configurations, respectively.

## 5. Experimental Results

**Figure 17:**
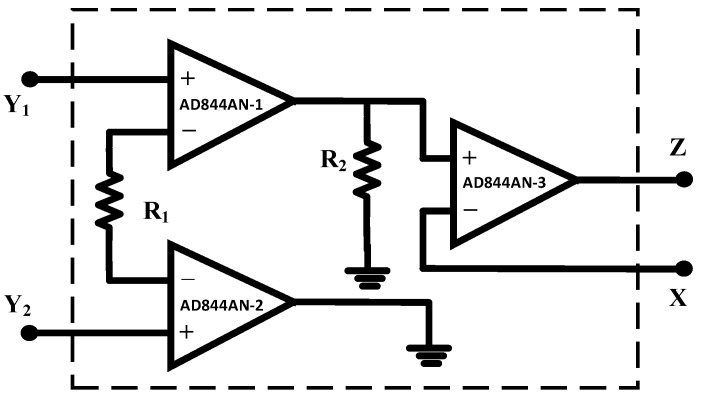
DVCC implementation using AD844AN.

**Figure 18:**
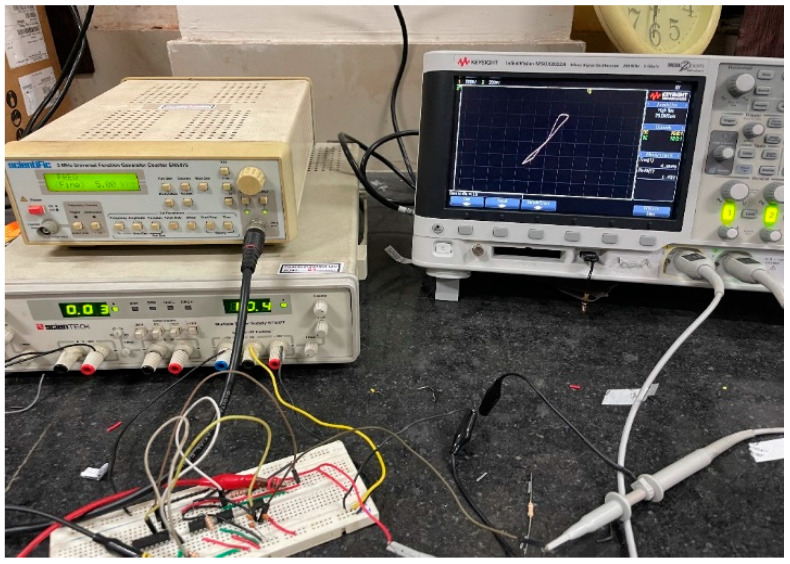
Complete experimental setup of the proposed memristor.

**Figure 19:**
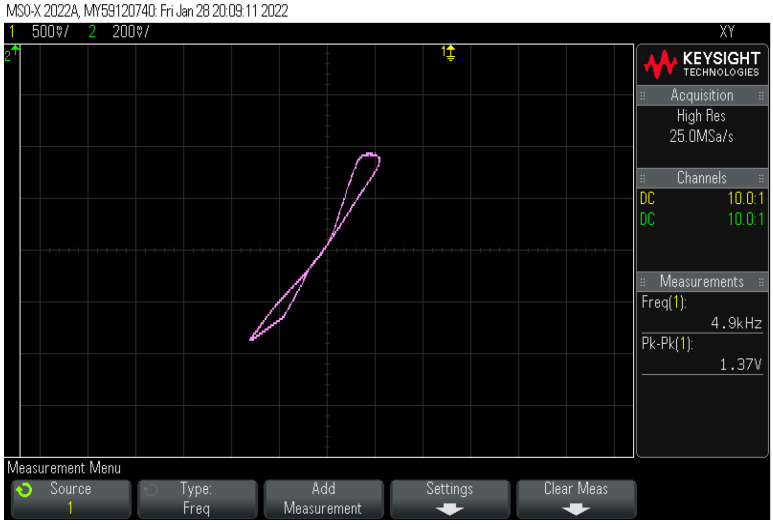
The experimental output of the proposed memristor at 5 kHz.

No commercial IC is available for DVCC, but it can be realized using AD844AN ICs. An AD844AN IC is a commercially available current feedback operational amplifier (CFOA) manufactured by Analog Devices. To establish the circuit connections of a DVCC, three AD844AN ICs are required. An IC-based DVCC-implemented circuit is shown in Figure 17. The experimental setup for the proposed memristor is implemented using three AD844AN and one CD4007 ICs. Among the available PMOS in the CD4007 IC pin configuration, we made use of a single PMOS from this IC. The supply voltage obtained for the experiments is ±10 V. The input signal has a 1.4 V peak at 5 kHz. The complete experimental setup is shown in Figure 18. The observed outcome is illustrated in Figure 19. 

## 6. Application

**Figure 20:**
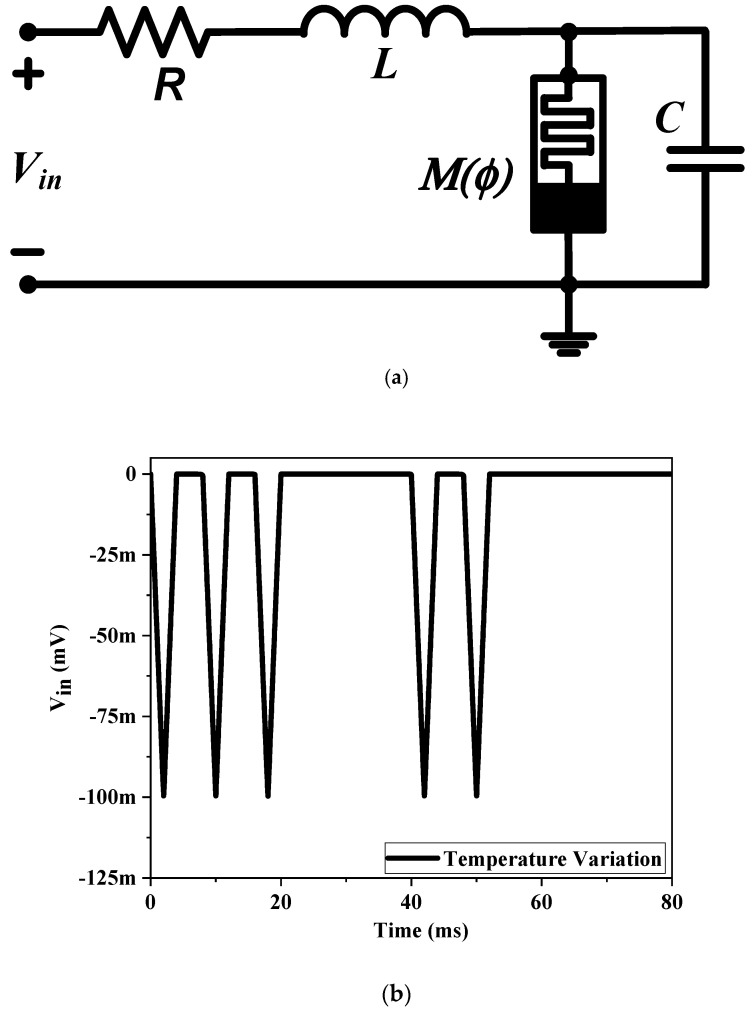

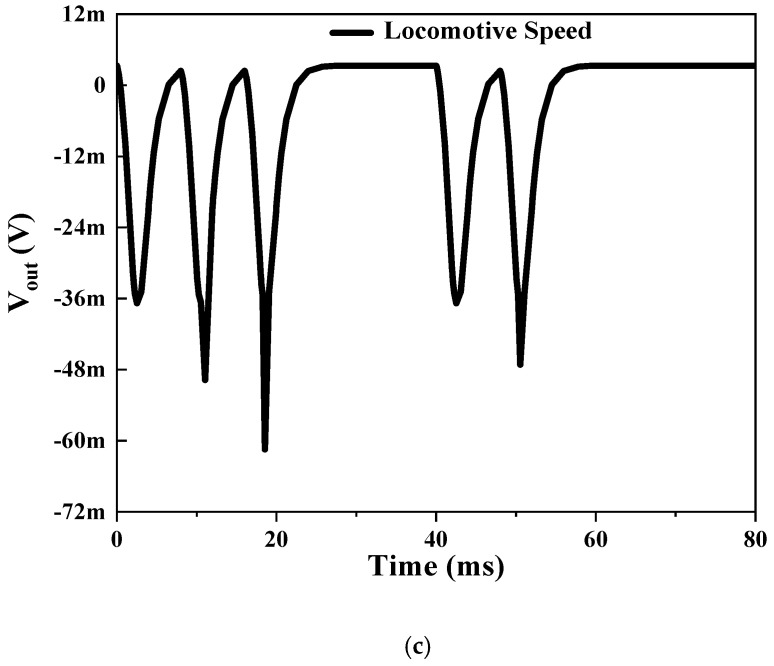
Proposed memristive model-based (**a**) adaptive amoeba-learning circuit (**b**) input voltage pulse (**c**) acquired speed response.

The memristive system can provide in-memory computing similar to that of the brain since it lacks a separate memory and processing unit. Neuromorphic computing has become one of the potential applications of memristive systems. The memristors store the information in the form of resistance. As a result, neuromorphic computing predominantly uses this memristor feature. The simplest eukaryotic life, an amoeba, has evolved a primitive nervous system. The decision-making capacity of the amoeba allows it to change its locomotive speed in response to changes in the surrounding environment’s temperature. The memristor-based simple RLC analogous model of the neuromorphic adaptive learning circuit [27] is derived from the behavioral response of amoeba. Using the proposed memristor and the RLC circuit, as illustrated in Figure 20a, it is possible to demonstrate the adaptive behavior of amoebas. The output voltage (*V_out_*) across the capacitor (*C*) corresponds to the amoeba’s locomotive speed, whereas the input voltage (*V_in_*) that drives the amoeba’s locomotion corresponds to the temperature and humidity. The inductor (*L*) and capacitor (*C*) store the energy in the form of magnetic and electric fields causing energy to shift from one form to another and do so oscillatory, which can result in resonance. In contrast, the parallel connection of the memristor stores the prior state. Resistance (*R*)-induced damping in the RLC circuit, which dictates its resonance nature, is utilized to simulate the movements of an amoeba using the oscillations of the stated RLC circuit. To anticipate the events observed for amoebas, a train of voltage signals was applied to the circuit. Resonance begins as the temperature and the variable resonance frequency (f) become equal. The applied temperature variation (*V_in_*) changes the memductance value until it meets the circuit resonance. The component values are considered to be *R* = 1 k, *L* = 10 mH, and 0.1 nF for the capacitor. As the temperature declines, the output indicating voltage proportional to locomotive speed decreases, as shown in Figure 20b,c. The temperature decreases many times during the first learning phase when the locomotive slows down, after which the motions become sluggish. Additionally, as a result of the amoeba’s capacity for learning, the locomotive speed begins to slow down immediately following a change in temperature starting with the next episode. As a result, from an application standpoint, the proposed memristor architecture is suitable for an adaptive learning circuit.

## 7. Summary

In this article, an attempt to design a 100 MHz high-frequency flux-controlled memristor emulator using only one DVCC was carried out. Along with a DVCC, three PMOS and one capacitor were used to design the memristor emulator with an added feature of external tunability. Even the transistor count was significantly low. The PVT and Monte Carlo simulations point to the robust design of the proposed emulator. Furthermore, the PHL obtained through simulation corroborates with the experimental result, thereby validating the theoretical aspects of the design. Henceforth, it can be inferred that this emulator is capable enough to find its use in various real-world applications such as in signal processing, chaotic circuits, communication systems, neural computations, etc. A memristor-based adaptive amoeba-learning circuit was also implemented to justify that the proposed circuit is suitable for real-world application. Although this design is suitable for various real-world applications, it cannot be used in applications requiring a floating memristor, as the proposed design implements only grounded configuration.

## Figures and Tables

**Table 1:** Comparison of proposed memristor with available designs.

Ref. & Year	Active Components	Power Supply (*V*)	No. of MOS	Passive Comp. (*R*, *C*)	Operating Freq. (Hz)	I/D *	Exp. Results	Tech. Used	Power Dissipation (W)
[9] 2017	1 CCII, 1 Multiplier	±10	-	1, 1	860 k	-	Yes	BJT	--
[12] 2017	1 MO-OTA, 1 Multiplier	±1.25/±5	>38	1, 1	1 k	-	Yes	CMOS/BJT	--
[13] 2018	2 OTA	±1.2	34	0, 1	8 M	Both	Yes	CMOS	--
[14] 2019	1 VDCC, 2 Transistors	±0.9	26	0, 1	2 M	Both	Yes	CMOS	--
[15] 2017	1 CBTA, 1 Multiplier	±0.9	23	2, 1	460 k	-	No	CMOS	--
[17] 2021	1 FB-VDBA	±0.9	19	0, 1	1 M	Both	No	CMOS	--
[18] 2018	1 VDTA	±0.9	16	0, 1	50 M	Both	Yes	CMOS	--
[19] 2020	1 VDTA	±0.9	16	1, 1	50 M	Both	Yes	CMOS	8 µ
[20] 2020	1 CDTA, 1 OTA	±0.9	36	0, 1	2 M	Both	No	CMOS	--
[22] 2021	1 CFTA	±1.2	28	0, 1	9 M	Both	Yes	CMOS	--
[23] 2020	1 CCII, 1 OTA	±1.2	24	1, 1	25.3 M	Both	Yes	CMOS	9.56 m
[24] 2020	1 CDBA, 1 OTA	±0.9	27	0, 1	1 M	Both	No	CMOS	--
[25] 2020	1 DO-OTA, 1 DVCC, 2 Transistors	±0.9	29	0, 1	1.5 M	I	Yes	CMOS	--
[26] 2021	1 VDCC, 1 OTA	--	35	2, 1	1 M	I	Yes	CMOS	--
[29] 2022	1 DVCC, 1 OTA	±0.9	23	1, 1	30 M	Both	Yes	CMOS	591 µ
[30] 2021	2 MVDCC	±0.9	52	2, 1	500 k	I	Yes	CMOS	--
[31] 2022	1 DVCCTA	±1	27	2, 1	12.8 M	Both	Yes	CMOS	8.74 m
[32] 2022	1 VDCC, 2 MOS	±0.9	24	0, 1	10 M	Both	No	CMOS	--
[32] 2022	2 VDCC, 2 MOS	±0.9	46	0, 1	50 M	Both	No	CMOS	--
Proposed Design	1 DVCC, 3 Transistors	±1.25	15	0, 1	100 M	Both	Yes	CMOS	7.64 µ

* I/D: Incremental/Decremental.

**Table 2:** Aspect Ratio of MOSFET used in DVCC.

MOSFET	*W* (in µm)	*L* (in nm)
M1–M4	5	360
M5–M6, M11–M12	4	360
M7–M9, M15	10	360
M13–M14	20	360
M10	9	360
